# Impact of Immune Checkpoint Inhibitors and Local Radical Treatment on Survival Outcomes in Synchronous Oligometastatic NSCLC

**DOI:** 10.1016/j.jtocrr.2025.100790

**Published:** 2025-01-08

**Authors:** Mandy Jongbloed, Valentina Bartolomeo, Martina Bortolot, Shahan Darwesh, Jarno W.J. Huijs, Safiye Dursun, Juliette Degens, Ben E.E.M. van den Borne, Maggy Youssef-El Soud, Marcel Westenend, Cordula Pitz, Dirk K.M. De Ruysscher, Lizza E.L. Hendriks

**Affiliations:** a Department of Pulmonary Diseases, GROW – Research Institute for Oncology and Reproduction, Maastricht University Medical Center, Maastricht, The Netherlands; b Radiation Oncology, Fondazione IRCCS Policlinico San Matteo, Pavia, Italy; c Department of Clinical Surgical, Diagnostic and Pediatric Sciences, Pavia University, Pavia, Italy; d Department of Radiation Oncology (Maastro Clinic), Maastricht University Medical Center, GROW – Research Institute for Oncology and Reproduction, Maastricht, The Netherlands; e Department of Medicine (DMED), University of Udine, Udine, Italy; f Department of Pulmonary Diseases, Zuyderland Hospital, Heerlen, The Netherlands; g Department of Pulmonary Diseases, Catharina Hospital, Eindhoven, The Netherlands; h Department of Pulmonary Diseases, Maxima Medical Center, Eindhoven, The Netherlands; i Department of Pulmonary Diseases, Viecuri hospital, Venlo, The Netherlands; j Department of Pulmonary Diseases, Laurentius hospital, Roermond, The Netherlands

**Keywords:** NSCLC, Synchronous oligometastatic disease, Immune checkpoint inhibitors, Progression free survival, Overall survival

## Abstract

**Introduction:**

The impact of an immune checkpoint inhibitor (ICI)–based systemic treatment strategy with or without local radical treatment (LRT) on outcomes for patients with NSCLC and synchronous oligometastatic disease (sOMD) is unknown.

**Methods:**

Multicenter retrospective study including adequately staged patients, with sOMD NSCLC (maximum five metastases in three organs [European Organization for Research and Treatment of Cancer definition]) between January 1, 2015 and December 31, 2022, treated with a first-line ICI-based versus chemotherapy-only regimen. Primary end points were progression-free survival and overall survival (OS) for an ICI-based versus chemotherapy-only strategy. Subgroup analyses were performed for patients who were deemed candidates for LRT in the multidisciplinary meeting and those proceeding to LRT.

**Results:**

A total of 416 patients were included, treated with chemotherapy-ICI (n = 138) or chemotherapy-only (n = 278), 319 out of 416 were deemed candidates by multidisciplinary meetings for LRT, whereas 192 (60%) proceeded to LRT. The median OS was significantly longer in the chemotherapy-ICI compared with the chemotherapy-only group (33.6 versus 15.9 mo, hazard ratio [HR] = 0.5, 95% confidence interval [CI]: 0.4–0.7, *p* < 0.001), in the subgroups who were candidate for LRT (36.1 versus 17.2 mo, HR = 0.5, 95% CI: 0.4–0.7, *p* < 0.001) and those proceeding to LRT (not reached versus 23.1 mo, HR = 0.4, 95% CI: 0.2–0.7, *p* < 0.001). In multivariate analysis, an ICI-based strategy was associated with improved survival in the total group (HR = 0.6, 95% CI: 0.4–0.9, *p* < 0.001), in those with intention of LRT (HR = 0.6, 95% CI: 0.4–0.9, *p* = 0.02) and those who proceeded to LRT (HR = 0.3, 95% CI: 0.1–0.6, *p* = 0.002).

**Conclusions:**

An ICI-based systemic treatment strategy (±LRT) is associated with improved survival compared with chemotherapy-only (±LRT) for patients with sOMD NSCLC. Prospective randomized trial data are necessary to identify patients most likely to benefit from adding LRT.

## Introduction

NSCLC is the leading cause of cancer-related mortality worldwide.^1^ Oligometastatic disease (OMD) is increasingly recognized as a subgroup within the metastatic spectrum. Hellman et al.^2^ defined oligometastases as a limited metastatic disease, potentially achieving long-term disease control or even cure when local radical therapy (LRT) is added. The interest in OMD is increasing because of the promising survival outcomes from small studies and advancements in LRT and diagnostic imaging.^3^, ^4^, ^5^, ^6^, ^7^, ^8^ Synchronous OMD (sOMD) is defined as OMD at initial diagnosis.^9^^,^^10^ For NSCLC, a clear definition of the maximum number of metastatic lesions allowed to be classified as OMD has been lacking, and staging requirements for clinical trials have been suboptimal. Fluorodeoxyglucose–positron emission tomography-computed tomography (FDG-PET-CT) and brain imaging with magnetic resonance imaging were often not mandated,^10^^,^^11^ leading to differences in patient selection for clinical trials and treatment strategies in daily practice, causing varying outcomes. Therefore, the European Organization for Research and Treatment of Cancer (EORTC) lung cancer group has established a consensus on the definition of sOMD NSCLC: after adequate staging (FDG-PET-CT and brain imaging) patients can have less than or equal to five metastases in less than or equal to three organs.^10^ Clinical guidelines advise adding LRT to systemic therapy to treat sOMD NSCLC.^12^, ^13^, ^14^ This is on the basis of limited evidence, coming from small randomized phase 2 trials and single-arm (retrospective) studies, with suboptimal staging and no intention-to-treat analysis.^3^, ^4^, ^5^, ^6^, ^7^^,^^15^, ^16^, ^17^ Most of them were performed before the introduction of immune checkpoint inhibitors (ICI), which could be beneficial for patients with sOMD.^15^^,^^18^^,^^19^ We previously performed a small hypothesis-generating retrospective single-center study comparing survival outcomes of patients with sOMD (EORTC definition) NSCLC treated with either first-line chemotherapy-ICI ([chemo-]ICI) (n=18) or chemotherapy-only (n = 50).^20^ Our intention-to-treat data suggested that an ICI-based strategy could be beneficial in terms of progression-free survival (PFS), but the patient number was limited and follow-up was short (19 mo) for patients treated with an ICI-based regimen. Overall survival (OS) data were not mature yet. To overcome these limitations, we updated our data set and expanded to a multicenter retrospective study. In addition, we evaluated clinical features associated with survival outcomes.

## Material and Methods

### Study Design and Patient Inclusion

This was a retrospective multicenter study in six Dutch hospitals, evaluating all patients discussed between January 1, 2015 and December 31, 2022 at the weekly thoracic oncology multidisciplinary meetings (MDTs) of these hospitals. In the Netherlands, all patients with newly diagnosed NSCLC have to be discussed at the MDT, so no patients could be missed.

Patients were included when they met the EORTC definition and staging criteria for sOMD NSCLC and were 18 years or older at the time of diagnosis.^10^ Patients had to be treated with first-line (chemo-)ICI or platinum-doublet chemotherapy regardless of whether they were planned to or actually received LRT to avoid selection bias. Depending on institutional policy, informed consent was signed by each patient still alive, and for deceased patients, informed consent was waived by the ethics committee. Patients were excluded when they had registered objections against the use of their data for scientific research. Additional exclusion criteria were the following: (1) second primary NSCLC; (2) no FDG-PET-CT scan at diagnosis; (3) no dedicated brain imaging at diagnosis (brain magnetic resonance imaging or diagnostic CT scan); (4) another malignancy within 5 years of NSCLC diagnosis (except tumor in-situ); (5) treatment with first-line tyrosine kinase inhibitor; (6) patients participating in a clinical trial or complete lack of follow-up data. Baseline characteristics collected from the digital medical files were the following: age, sex, weight at diagnosis, length, WHO performance status (WHO-PS), smoking status, data on histologic diagnosis biochemical data within 30 days before the start of treatment or at the date of the first pathologic confirmed NSCLC diagnosis, molecular and programmed death-ligand 1 (PD-L1) testing, TNM stage on the basis of the eighth edition of the American Joint Committee on Cancer including the number and location of metastases, maximum diameter of the primary tumor, the intent of the MDT whether the patient could have radical oligometastatic treatment, information on first-line systemic therapy, treatment toxicity (graded according to the Common Terminology Criteria for Adverse Events version 5.0), response after finishing induction systemic treatment (according to Response Evaluation Criteria in Solid Tumors 1.1), whether patients actually received LRT, date of disease progression, and date of last contact or death. This study was approved by Maastricht University Medical Center+ institutional review board (2021-2973) and the local institutional review boards of the participating hospitals.

In the participating centers, the same standard of care treatment protocol for sOMD was used: for fit patients, start systemic therapy (with or without upfront brain radiotherapy [RT] if deemed necessary), and after 3 to 4 months, a restaging and new MDT discussion to discuss LRT in case of disease response or no progression and a good clinical condition. All tumor sites of the patient had to be amenable for LRT, which consisted of surgery or radiation therapy to a biological dose (equivalent dose in 2 Gy fractions, α/β=8 Gy) of at least 55 Gy (except for brain metastases in which lower doses such as 24 Gy in three fractions were allowed). LRT of the primary tumor had to be either stereotactic body radiation therapy, more fractionated RT for central lesions, or lobectomy and a lobe-specific nodal dissection. More fractionated RT regimens, such as 55 Gy in 20 fractions or 66 Gy in 24 fractions, were used to treat mediastinal lymph nodes or centrally located tumors in patients with local stage T1-3 N0-1 disease. For extracranial distant metastases, radical resection or stereotactic body radiation therapy could be used as LRT. Patients with brain metastases could either be treated with resection followed by stereotactic radiotherapy (SRT) of the resection site or SRT of the brain up to 24 Gy in a single fraction on the basis of the location of the lesion. Both surgery and radiation therapy were allowed in the same patient. The decision for patients eventually proceeding to LRT after induction systemic therapy was made by an individual decision stated by the MDT (discussed by thoracic oncologists, cardiothoracic surgeons, radiation oncologists, and radiologists) and the decision was carefully considered on the basis of the performance status of the patient, response to induction systemic therapy, and the effects and toxicity of the proposed treatment strategy.

### Statistical Analysis

Statistical analysis was performed using IBM Statistical Package for the Social Sciences version 28 (IBM SPSS Statistics, IBM Corp., Armonk, New York). Baseline characteristics were analyzed by descriptive statistics and the groups were compared using the Mann-Whitney U-test and chi-square test. PFS and OS were calculated from the date of pathologic diagnosis and estimated using the Kaplan-Meier method. Patients without events were censored at the last follow-up date, and the median follow-up was estimated by the reversed Kaplan-Meier method. The PFS, OS, and median follow-up between the groups were compared using a Cox regression model. Univariate and multivariate analyses were performed using the Cox regression model. A *p* value of less than 0.05 was considered statistically significant.

### End Points

The primary end points were to estimate the effect of a (chemo-)ICI-based regimen on PFS and OS versus chemotherapy-only for all patients, and for the subgroup of patients who were treated with systemic therapy induction and candidate for LRT. In addition, the effect of adding LRT on PFS and OS after induction systemic therapy was estimated, stratified by patients who received (chemo-)ICI induction versus chemotherapy-only.

The secondary end points were Response Evaluation Criteria in Solid Tumors version 1.1 response on induction systemic therapy, the percentage of patients that proceeded to LRT, and the safety of adding LRT after induction systemic therapy ([chemo-]ICI versus chemotherapy-only).

## Results

### Baseline Characteristics of Patients

**Figure 1:**
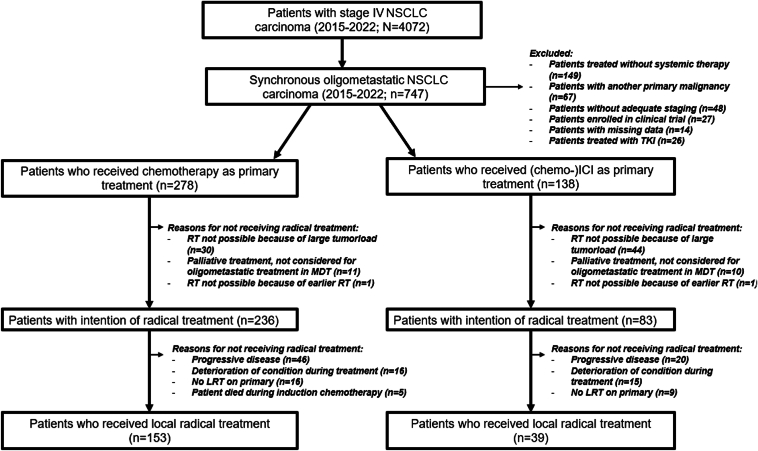
Flowchart of patient inclusion and selection with synchronous oligometastatic NSCLC. TKI, tyrosine kinase inhibitor; RT, radiotherapy; MDT, multidisciplinary team; ICI, immune checkpoint inhibitor; LRT, local radical treatment.

In total, 4072 patients with stage IV NSCLC were screened and 747 patients (18%) with sOMD NSCLC were identified between January 1, 2015 and December 31, 2022. Of these patients, 331 patients were excluded because of the following: (1) no systemic therapy because of poor clinical condition (n = 149); (2) another primary malignancy (n = 67); (3) no adequate staging (n = 48); (4) clinical trial participation (n = 27); (5) tyrosine kinase inhibitor treatment (n = 26); and (6) no follow-up because of subsequent treatment in another hospital (n = 14). Patient selection is depicted in Figure 1. Of the 416 included patients, 278 patients (67%) received chemotherapy-only and 138 patients (33%) received (chemo-)ICI as induction therapy (91/138 [66%] chemo-ICI and 47/138 [34%] ICI-monotherapy), the baseline characteristics are detailed in Table 1. In the MDT discussion at the presentation of sOMD NSCLC, the MDT advised LRT for 319 out of 416 patients (77%), 236 out of 278 patients (85%) in the chemotherapy-only group, and 83 out of 138 patients (60%) in the (chemo-)ICI group. Reasons to not advise LRT for 97 patients include the following: (1) tumor load too large for radical treatment (n = 74); (2) comorbidities preventing LRT (n = 21); or (3) no radical RT possible because of previous RT in the same field for a tumor treated at least 5 years before sOMD diagnosis (n = 2). Three patients had a WHO-PS of three at NSCLC diagnosis because of symptomatic cerebral edema from brain metastases, but their clinical condition rapidly improved after treatment with steroids and therefore were deemed candidates for LRT (Fig. 1). The details of the LRT strategy are described in Supplementary Table 1.

**Table 1:** Baseline Clinical Characteristics

Characteristics		(Chemotherapy)-ICI Group (n = 138)	Chemotherapy-Only Group (n = 278)	*p*-Value
Median age at diagnosis (range), y		67.0 (35–86)	67.0 (38–88)	0.99
Sex (%)				
	Male	68 (49)	153 (55)	0.27
	Female	70 (51)	125 (45)	0.27
WHO-PS (%)				
	0	80 (58)	134 (48)	0.06
	1	49 (36)	120 (43)	0.13
	2	9 (6)	21 (8)	0.70
	3^a^	0 (0)	3 (1)	0.22
BMI (range), kg/m^2^		24.9 (17.6–37.7)	24.6 (15.2–42.1)	0.66
Median LDH (range)		209 (107–699)	210 (99–645)	0.72
Median CRP (range), mg/L		17 (1–339)	17 (1–244)	0.50
Median serum albumin (range), g/dL		39.0 (25.0–49.0)	39.0 (23.0–53.0)	0.69
Median serum total protein, g/dL		74.0 (61.0–84.0)	73.0 (64.0–96.0)	0.87
Smoking status (%)				
	Current	58 (42)	117 (42)	0.99
	Former	77 (56)	152 (55)	0.83
	Never	3 (2)	9 (3)	0.54
NSCLC subtype (%)				
	Nonsquamous	105 (76)	214 (77)	0.84
	Squamous	33 (24)	64 (23)	0.84
PD-L1 status (%)				
	Positive (>50%)	70 (51)	53 (19)	**<0.001**
	Positive (1%–49%)	36 (26)	40 (15)	**0.004**
	Negative (<1%)	25 (18)	59 (21)	0.46
	Unknown	7 (5)	126 (45)	**<0.001**
Driver mutation^b^ (%)				
	ALK+	0 (0)	1 (0.4)	0.48
	BRAF+	2 (1)	4 (1)	0.99
	EGFR+	0 (0)	5 (2)	0.11
	HER2+	1 (1)	1 (0.4)	0.61
	KRAS+	44 (32)	75 (27)	0.30
Brain imaging	MRI brain	84 (61)	181 (65)	0.40
	Diagnostic CT of the brain	54 (39)	97 (35)	0.40
T stage^c^ (%)				
	x	4 (3)	11 (4)	0.59
	1a	1 (1)	5 (2)	0.39
	1b	7 (5)	17 (6)	0.67
	1c	9 (6)	19 (7)	0.91
	2a	20 (14)	34 (12)	0.52
	2b	11 (8)	26 (9)	0.64
	3	23 (17)	65 (24)	0.11
	4	63 (46)	101 (36)	0.07
N stage (%)				
	0	31 (23)	60 (22)	0.84
	1	8 (6)	25 (9)	0.26
	2	46 (33)	122 (44)	**0.04**
	3	53 (38)	71 (25)	**0.01**
Maximum diameter primary tumor (cm)		4.7 (0–11.0)	4.5 (0–13.6)	0.30
Median number of metastases		2	1	**<0.001**
Number of metastases (%)	1	47 (34)	158 (57)	**<0.001**
	2	48 (35)	75 (27)	0.10
	3	20 (14)	29 (11)	0.23
	4	14 (10)	9 (2)	**0.004**
	5	9 (7)	7 (3)	0.05
Metastatic sites (%)				
	Adrenal	24 (17)	51 (18)	0.81
	Bone	38 (28)	66 (24)	0.40
	Brain	31 (23)	91 (33)	**0.03**
	Liver	12 (9)	12 (4)	0.07
	Lung	42 (30)	68 (25)	0.19
	Nodal extrathoracic	29 (21)	34 (12)	**0.02**
	Pleural	9 (7)	12 (4)	0.33
	Other site^d^	10 (7)	16 (6)	0.55
Best response after induction systemic treatment according to RECIST 1.1 (%)				
	CR	2 (1)	6 (2)	0.59
	PR	68 (49)	118 (42)	0.28
	SD	45 (33)	94 (34)	0.65
	PD	20 (15)	46 (17)	0.51
	Unknown	3 (2)	14 (5)	0.62

*Note:p* values in bold are statistically significant.

AJCC, American Joint Committee on Cancer; BMI, body mass index; CR, complete response; CRP, C-reactive protein; CT, computed tomography; ICI, immune checkpoint inhibitor; LDH, lactate dehydrogenase; LRT, local radical treatment; MDT, multidisciplinary meeting; NGS, next-generation sequencing; PD-L1, programmed death-ligand 1; PD, progressive disease; PR, partial response; RECIST 1.1, Response Evaluation Criteria in Solid Tumors 1.1; SD, stable disease; TKI, tyrosine kinase inhibitor; WHO-PS, WHO Performance Score.

a Three patients had a WHO-PS of 3 due to symptomatic cerebral edema, but their clinical condition rapidly improved after treatment and were deemed candidates for LRT by the MDT.

b Not all driver mutations could be detected during the inclusion period and first-line TKI was not available yet for some patients. The patient with ALK translocation started chemotherapy before NGS result and therefore did not receive first-line TKI. Four patients with an EGFR mutation had an exon20 insertion mutation, and the one patient with an EGFR exon 19 deletion had a squamous cell carcinoma and was initially not tested for actionable genomic alterations.

c TNM staging is based on TNM 8 (AJCC). If staging was based on TNM 7 in the MDT, it is recalculated into TNM 8.

d Other sites: bowel, breast, pancreatic gland, peritoneal, soft tissue, spleen, subcutis, thyroid gland and kidney.

Significantly more patients with tumors with high PD-L1 expression, N3 disease, four metastases, and extrathoracic nodal metastases were in the (chemo-)ICI compared with the chemotherapy-only group (Table 1). Patients in the chemotherapy-only group had significantly more unknown PD-L1 expression (reflecting the time period in which chemotherapy-only was given), N2 disease, solitary metastasis, and brain metastases.

### Treatment Strategy

Eventually, 192 out of 319 patients (60%) of the patients who were initially by the MDT deemed candidates for LRT proceeded to LRT after a response to induction systemic therapy, and three additional patients had a long-lasting complete disease response after induction treatment (i.e., no LRT possible anymore as no visible disease); for analyses, these were also included in the LRT group (Table 2). Reasons for not proceeding to LRT were the following: (1) progressive disease after induction systemic therapy ([chemo]-ICI, n = 20; chemotherapy-only, n = 46); (2) deterioration of the clinical condition during induction therapy resulting in no response evaluation ([chemo]-ICI, n = 15; chemotherapy-only, n = 16); (3) no radical RT or surgery possible on primary tumor because of no response (stable disease) on induction systemic therapy ([chemo]-ICI, n = 9; chemotherapy-only, n = 16); and (4) five patients died during induction chemotherapy (Fig. 1). A significantly higher percentage of patients treated with chemotherapy-only proceeded to LRT (65% versus 47%, *p* = 0.002) (Table 2). The three patients with a complete response (CR) had induction with chemo-ICI and did not receive LRT, but because of the CR, they were counted as receiving actual radical treatment. Most of the patients not proceeding to LRT in the (chemo-)ICI group had more deterioration of the condition during treatment (18% versus 7%) compared with the patients treated with induction chemotherapy-only.

**Table 2:** Intention of Radical Treatment, Actual Radical Treatment and LRT

Treatment Strategy	(Chemotherapy)-ICI Group (n = 138)	Chemotherapy-Only Group (n = 278)	*p*-Value
MDT intention of radical treatment (% of total group)	83 (60)	236 (85)	**<0.001**
Actual radical treatment^a^ (% of patients with intention radical treatment)	39 (47)	153 (65)	**0.004**
LRT (% of patients with intention radical treatment)	37 (45)	152 (64)	**0.002**
LRT radiation therapy only (% of patients with LRT)	31 (84)	106 (70)	0.09
LRT surgery only (% of patients with LRT)	0 (0)	1 (1)	0.62
LRT combination radiation therapy and surgery (% of patients with LRT)	6 (16)	45 (30)	0.10
Palliative treatment (% of total group)	101 (72)	127 (45)	**0.004**

*Note:p* values in bold are statistically significant.

ICI, immune checkpoint inhibitor; MDT, multidisciplinary thoracic team; LRT, local radical therapy.

a Patients who were counted as actual radical treatment are the patients who received local radical treatment or those who had complete response after induction systemic therapy and did not receive LRT (n = 3).

### Safety Profile

Treatment-related adverse event (TRAE) including grade 3 or higher TRAE were similar and manageable in both groups (Supplementary Tables 2 and 3).

### Survival Analysis

The median follow-up for the whole cohort was 54.6 months (95% confidence interval [CI]: 48.0–61.1), the median PFS was 8.5 months (95% CI: 7.3–9.6) and the median OS was 19.1 months (95% CI: 16.2-22.0).

**Figure 2:**
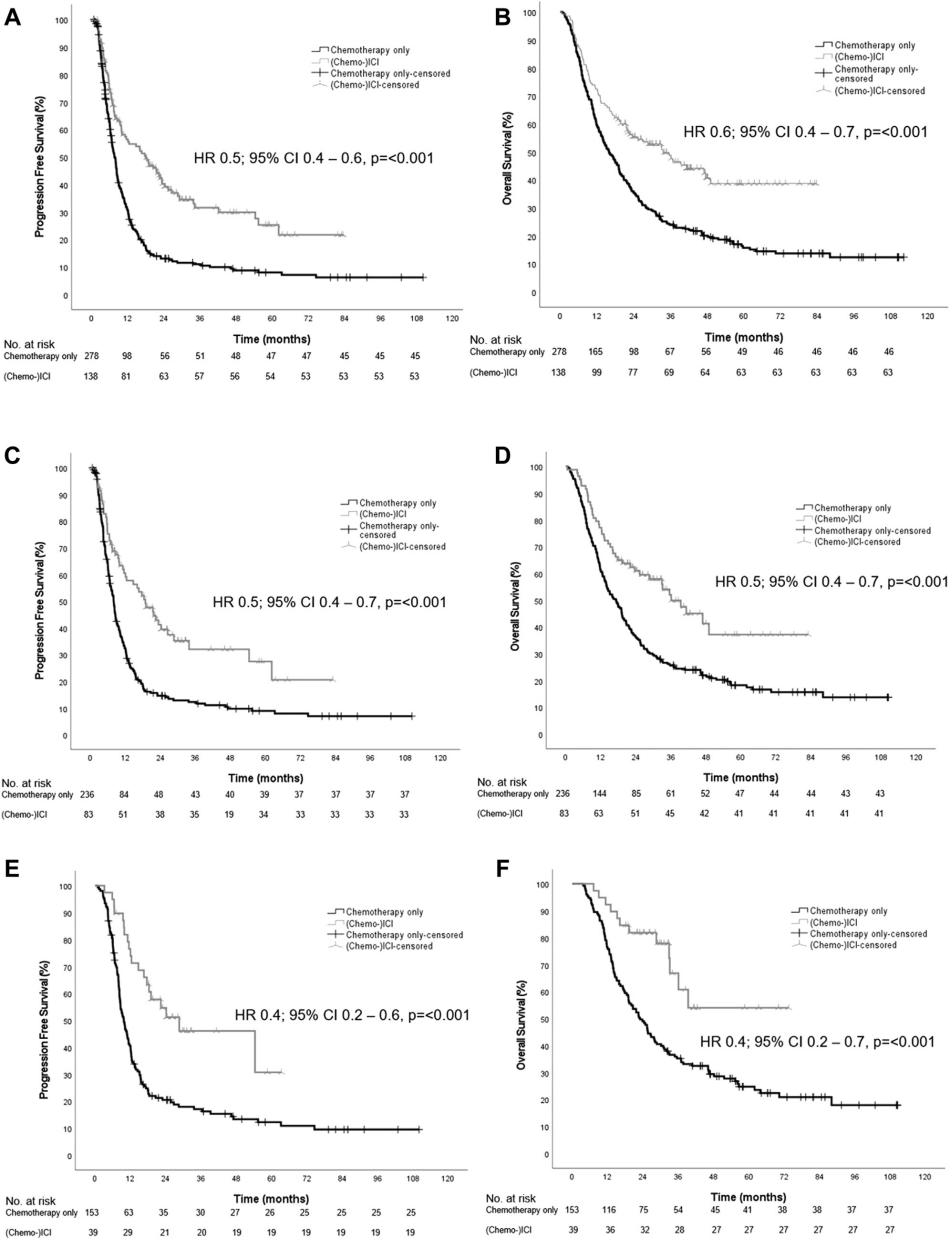
(*A*) Progression-free survival in the total group, split for (chemo)-ICI versus chemo-only treatment. (*B*) Overall survival in the total group, split for (chemo)-ICI versus chemo-only treatment. (*C*) Progression-free survival in the subgroup of patients with the intention of local radical therapy, split for (chemo)-ICI versus chemo-only treatment. (*D*) Overall survival in the subgroup of patients with the intention of local radical therapy, split for (chemo)-ICI versus chemo-only treatment. (*E*) Progression-free survival in the subgroup of patients actually receiving local radical therapy, split for (chemo)-ICI versus chemo-only treatment. (*F*) Overall survival in the subgroup of patients actually receiving local radical therapy, split for (chemo)-ICI versus chemo-only treatment. chemo, chemotherapy; CI, confidence interval; HR, hazard ratio; ICI, immune checkpoint inhibitor.

The median follow-up for the (chemo-)ICI group was significantly shorter than for the chemotherapy-only group: 42.6 (95% CI: 37.9–47.3) versus 66.4 months (95% CI: 50.0–82.8) (*p <* 0.001). A total of 85 patients (62%) in the (chemo-)ICI group had disease progression and 75 patients (54%) died, whereas these numbers were 233 (84%) and 233 (84%), respectively in the chemotherapy-only group. The median PFS and the median OS were significantly longer in the (chemo-)ICI compared with the chemotherapy-only group. The median PFS was 17.9 (95% CI: 10.5–25.3) versus 7.6 months (95% CI: 6.7–8.5) (hazard ratio [HR] = 0.5, 95% CI: 0.4–0.6) (Fig. 2*A*), and the median OS was 33.6 (95% CI: 20.0–47.2) versus 15.9 months (95% CI: 12.9–19.0) (HR = 0.6, 95% CI: 0.4–0.7, *p* < 0.001) (Fig. 2*B*).

The median PFS and the median OS were also significantly longer for (chemo-)ICI versus chemotherapy-only in the subgroup of patients with an MDT intention of LRT. The median PFS was 18.8 (95% CI: 12.2–25.5) versus 8.0 months (95% CI: 7.1–8.9) (HR = 0.5, 95% CI: 0.4–0.7, *p* < 0.001) (Fig. 2*C*), the median OS was 36.1 months (95% CI: 24.8–47.4) versus 17.2 (95% CI: 14.0–20.4) (HR = 0.5, 95% CI: 0.4–0.7, *p* < 0.001) (Fig. 2*D*).

The PFS and OS benefit in the (chemo-)ICI group compared with the chemotherapy-only group remained in the subgroup of patients actually receiving LRT, with a median PFS of 28.6 (95% CI: 5.7–51.5) versus 9.8 months (95% CI: 8.2–11.4) (HR = 0.4, 95% CI: 0.2–0.6, *p* < 0.001) and a median OS of not reached (95% CI: 22.1–33.4) versus 23.1 months (95% CI: 18.6–27.6) (HR = 0.4, 95% CI: 0.2–0.7, *p* < 0.001) (Fig. 2*E* and *F*).

In the multivariate analysis for the whole group, ICI-based treatment was associated with longer PFS whereas PD-L1 less than 1%, presence of adrenal gland or bone metastases, elevated levels of serum C-reactive protein (CRP) at diagnosis, and no response to induction systemic therapy were associated with a shorter PFS (Table 3). The presence of brain metastases, unknown PD-L1 status, elevated levels of CRP, and no response to induction systemic therapy were independently associated with shorter OS, and ICI treatment was independently associated with longer OS (Table 4).

**Table 3:** Cox Regression Analysis for Progression-Free Survival for All Patients

Characteristics		Univariate Analysis		Multivariate Analysis	
		Hazard Ratio (95% CI)	*p*-Value	Hazard Ratio (95% CI)	*p*-Value
Age (ref: <75 y)	≥75 y	1.1 (0.8–1.5)	0.71	0.8 (0.5–1.1)	0.21
Sex (ref: male)	Female	0.7 (0.6–1.0)	**0.04**	1.0 (0.8–1.3)	0.99
WHO-PS (ref: 0–1)	2–3	1.2 (0.8–1.7)	0.51	1.3 (0.8–2.1)	0.36
Smoking (ref: never)	Former	1.0 (0.8–1.2)	0.84		
	Current	1.5 (0.8–3.0)	0.16		
PD-L1 status (ref: >50% expression)	1%–49% expression	1.4 (1.0–2.0)	**0.04**	1.1 (0.7–1.4)	0.56
	<1% expression	2.1 (1.5–2.9)	**<0.001**	1.7 (1.1–2.4)	**0.01**
	Unknown	1.8 (1.3–2.4)	**<0.001**	1.0 (0.7–1.4)	0.98
Histology (ref: nonsquamous)	Squamous	0.9 (0.7–1.2)	0.58		
Brain imaging (ref: MRI)	Diagnostic CT-scan	1.1 (0.8–1.3)	0.71		
T stage (ref: 1–2)	3–4	1.3 (1.0–1.6)	0.05		
N stage (ref: 0–1)	2–3	1.1 (0.9–1.4)	0.42		
Total metastases (ref: 1)	>1	1.2 (0.9–1.4)	0.26		
Metastatic sites					
(ref: no adrenal gland)	Adrenal gland	1.5 (1.1–1.9)	**0.01**	1.5 (1.0–2.0)	**0.03**
(ref: no bone)	Bone	1.4 (1.1–1.8)	**0.01**	1.6 (1.2–2.2)	**0.003**
(ref: no brain)	Brain	0.9 (0.7–1.1)	0.45		
(ref: no extrathoracic nodal)	Extrathoracic nodal	0.9 (0.7–1.2)	0.47		
(ref: no lung)	Lung	0.8 (0.6–1.0)	0.05		
(ref: no other^a^)	Other site	1.4 (1.1–1.9)	**0.02**	1.4 (1.0–2.0)	**0.03**
Serum albumin (ref: <40 g/L)	≥40 g/L	0.8 (0.6–1.1)	0.10		
Serum CRP (ref: ≤5mg/L)	>5 mg/L	1.8 (1.3–2.5)	**<0.001**	2.0 (1.5–2.8)	**<0.001**
Serum LDH (ref: <248)	≥248	0.8 (0.6–1.1)	0.18		
Best response to induction systemic therapy (ref: CR and PR)	SD and PD	2.4 (1.9–3.0)	**<0.001**	2.6 (2.0–3.4)	**<0.001**
Immunotherapy (ref: No)	Yes	0.5 (0.4–0.6)	**<0.001**	0.5 (0.4–0.7)	**<0.001**
TRAE (ref: toxicity ≤2)	Toxicity grade ≥3	1.3 (0.7–2.3)	0.42		

*Note:p* values in bold are statistically significant.

CI, confidence interval; CR, complete response; CRP, C-reactive protein; CT, computed tomography; LDH, lactate dehydrogenase; MRI, magnetic resonance imaging; PD-L1, programmed death-ligand 1; PD, progressive disease; PR, partial response; Ref, reference; SD, stable disease; TRAE, treatment related adverse event; WHO-PS, WHO Performance Score.

a Bowel, breast, liver, pancreatic gland, peritoneal, pleural, soft tissue, spleen, subcutis, thyroid gland and renal.

**Table 4:** Cox Regression Analysis for Overall Survival for All Patients

Characteristics		Univariate Analysis		Multivariate Analysis	
		Hazard Ratio (95% CI)	*p*-Value	Hazard Ratio (95% CI)	*p*-Value
Age (ref: <75 y)	≥75 y	1.4 (1.0–1.8)	**0.04**	1.3 (0.9–1.9)	0.13
Sex (ref: male)	Female	0.7 (0.6–0.99)	**0.04**	0.8 (0.6–1.1)	0.23
WHO-PS (ref: 0–1)	2–3	1.7 (1.2–2.5)	**0.01**	1.6 (0.9–2.5)	0.07
Smoking (ref: never)	Former	0.8 (0.4–1.6)	0.55		
	Current	0.9 (0.4–1.6)	0.63		
PD-L1 status (ref: >50% expression)	1%–49% expression	1.6 (1.2–2.3)	**<0.001**	1.4 (0.9–2.1)	0.14
	<1% expression	1.7 (1.2–2.4)	**0.01**	1.1 (0.7–1.6)	0.67
	Unknown	2.1 (1.5–2.8)	**<0.001**	1.6 (1.0–2.3)	**0.02**
Histology (ref: nonsquamous)	Squamous	0.9 (0.7–1.2)	0.46		
Brain imaging (ref: MRI)	Diagnostic CT-scan	1.0 (0.8–1.3)	0.82		
T stage (ref: 1–2)	3–4	1.2 (0.9–1.5)	0.15		
N stage (ref: 0–1)	2–3	1.0 (0.8–1.3)	0.96		
Total metastases (ref: 1)	>1	1.1 (0.9–1.4)	0.37		
Metastatic sites					
(ref: no adrenal gland)	Adrenal gland	1.5 (1.1–1.9)	**0.01**	1.4 (0.9–1.9)	0.08
(ref: no bone)	Bone	1.5 (1.2–2.0)	**0.002**	1.3 (1.0–1.8)	0.07
(ref: no brain)	Brain	1.4 (0.6–0.9)	**0.01**	1.5 (1.5–2.1)	**0.03**
(ref: no extrathoracic nodal)	Extrathoracic nodal	0.9 (0.6–1.2)	0.33		
(ref: no lung)	Lung	1.0 (0.8–1.3)	0.90		
(ref: no other^a^)	Other site	0.9 (0.6–1.1)	0.27		
Serum albumin (ref: <40 g/L)	≥40 g/L	0.8 (0.6–1.1)	0.19		
Serum CRP (ref: ≤5 mg/L)	>5 mg/L	2.1 (1.5–2.9)	**<0.001**	2.1 (1.5–2.9)	**<0.001**
Serum LDH (ref: <248)	≥248	0.9 (0.7–1.2)	0.46		
Best response to induction systemic therapy (ref: CR and PR)	SD and PD	2.6 (2.1–3.3)	**<0.001**	2.7 (2.0–3.6)	**<0.001**
Immunotherapy (ref: no)	Yes	0.6 (0.4–0.7)	**<0.001**	0.6 (0.4–0.9)	**0.004**
TRAE (ref: toxicity ≤2)	Toxicity grade ≥3	1.4 (0.8–2.7)	0.26		

*Note:p* values in bold are statistically significant.

CI, confidence interval; CR, complete response; CRP, C-reactive protein; CT, computed tomography; LDH, lactate dehydrogenase; MRI, magnetic resonance imaging; PD-L1, programmed death-ligand 1; PD, progressive disease; PR, partial response; Ref, reference; SD, stable disease; TRAE, treatment related adverse event; WHO-PS, WHO Performance Score.

a Bowel, breast, liver, pancreatic gland, peritoneal, pleural, soft tissue, spleen, subcutis, thyroid gland and renal.

In multivariate analysis for PFS in the subgroup of patients with the intention of radical treatment, ICI use was associated with better survival and PD-L1 less than 1%, presence of bone metastases, elevated level of serum CRP, and no response to induction systemic therapy were associated with poor survival (Table 5). ICI use was associated with longer OS in multivariate analysis and the presence of brain metastases, elevated level of serum CRP, and no response to induction systemic therapy were associated with shorter OS (Table 6).

**Table 5:** Cox Regression Analysis for Progression-Free Survival for All Patients With the Intention of Receiving Radical Treatment

Characteristics		Univariate Analysis		Multivariate Analysis	
		Hazard Ratio (95% CI)	*p*-Value	Hazard Ratio (95% CI)	*p*-Value
Age (ref: <75 y)	≥75 y	1.1 (0.8–1.6)	0.62	1.2 (0.7–1.9)	0.47
Sex (ref: male)	Female	0.7 (0.6–0.9)	**0.01**	0.9 (0.7–1.3)	0.66
WHO-PS (ref: 0–1)	2–3	1.2 (0.7–2.0)	0.46	1.2 (0.6–2.4)	0.53
Smoking (ref: never)	Former	1.1 (0.8–1.4)	0.53		
	Current	1.8 (0.9–3.5)	0.11		
PD-L1 status (ref: >50% expression)	1%–49% expression	1.2 (0.8–1.8)	0.29	1.4 (0.9–2.3)	0.20
	<1% expression	1.9 (1.3–2.8)	**<0.001**	1.7 (1.1–2.6)	**0.03**
	Unknown	1.5 (1.1–2.1)	**0.02**	1.1 (0.7–1.6)	0.81
Histology (ref: nonsquamous)	Squamous	1.0 (0.7–1.3)	0.74		
Brain imaging (ref: MRI)	Diagnostic CT-scan	1.2 (0.9–1.5)	0.26		
T Stage (ref: 1–2)	3–4	1.2 (1.0–1.6)	0.11		
N Stage (ref: 0–1)	2–3	1.2 (0.9–1.6)	0.23		
Total metastases (ref: 1)	>1	1.1 (0.9–1.4)	0.43		
Metastatic sites					
(ref: no adrenal gland)	Adrenal gland	1.7 (1.2–2.4)	**0.002**	1.4 (0.9–2.2)	0.13
(ref: no bone)	Bone	1.5 (1.1–2.1)	**0.01**	1.7 (1.1–2.5)	**0.01**
(ref: no brain)	Brain	0.9 (0.7–1.2)	0.36		
(ref: no extrathoracic nodal)	Extrathoracic nodal	0.9 (0.6–1.2)	0.43		
(ref: no lung)	Lung	0.7 (0.5–0.9)	**0.01**	0.7 (0.5–1.1)	0.12
(ref: no other^a^)	Other site	1.6 (1.1–2.2)	**0.01**	1.3 (0.8–2.0)	0.18
Serum albumin (ref: <40 g/L)	≥40 g/L	0.8 (0.6–1.2)	0.34		
Serum CRP (ref: ≤5 mg/L)	>5 mg/L	1.6 (1.1–2.3)	**0.01**	1.8 (1.3–2.7)	**0.002**
Serum LDH (ref: <248)	≥248	0.9 (0.7–1.2)	0.43		
Best response to induction systemic therapy (ref: CR and PR)	SD and PD	2.4 (1.9–3.2)	**<0.001**	2.4 (1.8–3.3)	**<0.001**
Immunotherapy (ref: No)	Yes	0.5 (0.4–0.7)	**<0.001**	0.5 (0.3–0.8)	**<0.001**
TRAE (ref: toxicity ≤ 2)	Toxicity grade ≥3	1.5 (0.8–2.9)	0.20		

*Note:p* values in bold are statistically significant.

CI, confidence interval; CR, complete response; CRP, C-reactive protein; LDH, lactate dehydrogenase; CT, computed tomography; MRI, magnetic resonance imaging; PD-L1, programmed death-ligand 1; PD, progressive disease; PR, partial response; Ref, reference; SD, stable disease; TRAE, treatment related adverse event; WHO-PS, WHO Performance Score.

a Bowel, breast, liver, pancreatic gland, peritoneal, pleural, soft tissue, spleen, subcutis, thyroid gland and renal.

**Table 6:** Cox Regression Analysis for Overall Survival for all Patients With the Intention of Receiving Radical Treatment

Characteristics		Univariate Analysis		Multivariate Analysis	
		Hazard Ratio (95% CI)	*p*-Value	Hazard Ratio (95% CI)	*p*-Value
Age (ref: <75 y)	≥75 y	1.6 (1.1–2.2)	**0.01**	1.5 (1.0–2.4)	0.06
Sex (ref: male)	Female	0.6 (0.5–0.8)	**<0.001**	0.9 (0.6–1.3)	0.58
WHO-PS (ref: 0–1)	2–3	1.8 (1.1–2.8)	**0.02**	1.5 (0.8–2.9)	0.19
Smoking (ref: never)	Former	0.7 (0.3–1.3)	0.27		
	Current	0.7 (0.4–1.5)	0.38		
PD-L1 status (ref: >50% expression)	1%–49% expression	1.3 (0.9–2.0)	0.20	1.3 (0.8–2.1)	0.39
	<1% expression	1.7 (1.2–2.4)	**0.01**	1.1 (0.7–1.7)	0.79
	Unknown	1.9 (1.4–2.7)	**<0.001**	1.5 (1.0–2.3)	0.06
Histology (ref: nonsquamous)	Squamous	0.9 (0.7–1.2)	0.59		
Brain imaging (ref: MRI)	Diagnostic CT-scan	1.1 (0.8–1.4)	0.70		
T Stage (ref: 1-2)	3–4	1.1 (0.8–1.4)	0.58		
N Stage (ref: 0-1)	2–3	1.0 (0.8–1.3)	0.94		
Total metastases (ref: 1)	>1	1.0 (0.8–1.3)	0.85		
Metastatic sites					
(ref: no adrenal gland)	Adrenal gland	1.5 (1.1–2.2)	**0.02**	1.5 (0.9–2.2)	0.10
(ref: no bone)	Bone	1.7 (1.3–2.3)	**<0.001**	1.4 (0.9–2.0)	0.14
(ref: no brain)	Brain	1.6 (0.2–2.1)	**0.002**	1.7 (1.1–2.5)	**0.02**
(ref: no extrathoracic nodal)	Extrathoracic nodal	0.9 (0.6–1.3)	0.63		
(ref: no lung)	Lung	0.9 (0.7–1.2)	0.44		
(ref: no other^a^)	Other site	1.4 (0.9–2.0)	0.06		
Serum albumin (ref: <40 g/L)	≥40 g/L	1.1 (0.9–1.6)	0.57		
Serum CRP (ref: ≤5 mg/L)	>5 mg/L	1.9 (1.3–2.8)	**<0.001**	1.8 (1.2–2.7)	**0.004**
Serum LDH (ref: <248)	≥248	1.0 (0.7–1.3)	0.89		
Best response to induction systemic therapy (ref: CR and PR)	SD and PD	2.5 (1.9–3.2)	**<0.001**	2.5 (1.8–3.5)	**<0.001**
Immunotherapy (ref: No)	Yes	0.5 (0.4–0.7)	**<0.001**	0.6 (0.4–0.9)	**0.02**
TRAE (ref: toxicity ≤ 2)	Toxicity grade ≥3	1.7 (0.9–3.6)	0.13		

*Note:p* values in bold are statistically significant.

CI, confidence interval; CR, complete response; CRP, C-reactive protein; CT, computed tomography; LDH, lactate dehydrogenase; MRI, magnetic resonance imaging; PD-L1, programmed death-ligand 1; PD, progressive disease; PR, partial response; Ref, reference; SD, stable disease; TRAE, treatment related adverse event; WHO-PS, WHO Performance Score.

a Bowel, breast, liver, pancreatic gland, peritoneal, pleural, soft tissue, spleen, subcutis, thyroid gland and renal.

In multivariate analysis for PFS in the subgroup of patients who received LRT, the presence of lung metastases and ICI-based regimen were associated with better survival, and PD-L1 less than 1% and an elevated level of serum CRP was associated with poor survival (Table 7). ICI-based regimen was associated with longer OS in multivariate analysis and an elevated level of serum CRP and no response to induction systemic therapy were associated with shorter OS (Table 8).

**Table 7:** Cox Regression Analysis for Progression-Free Survival for Patients Who Received LRT

Characteristics		Univariate Analysis		Multivariate Analysis	
		Hazard Ratio (95% CI)	*p*-Value	Hazard Ratio (95% CI)	*p*-Value
Age (ref: <75 y)	≥75 y	0.8 (0.5–1.4)	0.46	0.6 (0.3–1.1)	0.09
Sex (ref: male)	Female	0.7 (0.5–1.0)	0.07	0.9 (0.6–1.3)	0.62
WHO-PS (ref: 0–1)	2–3	1.9 (1.0–3.6)	0.06	1.2 (0.6–2.5)	0.69
Smoking (ref: never)	Former	1.1 (0.8–1.5)	0.71		
	Current	2.0 (0.8–5.0)	0.14		
PD-L1 status (ref: >50% expression)	1%–49% expression	1.4 (0.9–2.2)	0.09	1.6 (0.9–2.9)	0.15
	<1% expression	2.2 (1.3–3.5)	**0.002**	2.0 (1.1–3.6)	**0.02**
	Unknown	1.2 (0.8–1.9)	0.44	1.2 (0.7–2.0)	0.53
Histology (ref: nonsquamous)	Squamous	0.7 (0.7–1.6)	0.70		
Brain imaging (ref: MRI)	Diagnostic CT-scan	1.3 (0.9–1.8)	0.20		
T Stage (ref: 1–2)	3–4	1.3 (0.9–1.7)	0.18		
N Stage (ref: 0–1)	2–3	1.5 (0.8–2.8)	0.19		
Total metastases (ref: 1)	>1	0.9 (0.7–1.3)	0.61		
Metastatic sites					
(ref: no adrenal gland)	Adrenal gland	1.5 (0.9–2.5)	0.13		
(ref: no bone)	Bone	1.4 (0.9–2.2)	0.16		
(ref: no brain)	Brain	1.1 (0.8–1.5)	0.69		
(ref: no extrathoracic nodal)	Extrathoracic nodal	0.7 (0.5–1.2)	0.18		
(ref: no lung)	Lung	0.7 (0.5–1.0)	**0.03**	0.6 (0.4–0.9)	**0.02**
(ref: no other^a^)	Other site	1.7 (1.0–2.7)	**0.04**	1.6 (0.9–3.1)	0.12
Serum albumin (ref: <40 g/L)	≥40 g/L	0.9 (0.5–1.5)	0.71		
Serum CRP (ref: ≤5 mg/L)	>5 mg/L	1.7 (1.1–2.6)	**0.03**	1.9 (1.2–3.1)	**0.01**
Serum LDH (ref: <248)	≥248	0.9 (0.6–1.3)	0.44		
Best response to induction systemic therapy (ref: CR and PR)	SD	1.7 (1.2–2.3)	**0.003**	1.3 (0.9–2.1)	0.21
Immunotherapy (ref: No)	Yes	0.4 (0.2–0.6)	**<0.001**	0.3 (0.2–0.6)	**<0.001**
TRAE (ref: toxicity ≤ 2)	Toxicity grade ≥3	1.0 (0.7–1.4)	0.96		

*Note:p* values in bold are statistically significant.

CI, confidence interval; CR, complete response; CRP, C-reactive protein; CT, computed tomography; LDH, lactate dehydrogenase; LRT, local radical therapy; MRI, magnetic resonance imaging; PD-L1, programmed death-ligand 1; PD, progressive disease; PR, partial response; Ref, reference; SD, stable disease; TRAE, treatment related adverse event; WHO-PS, WHO Performance Score.

a Bowel, breast, liver, pancreatic gland, peritoneal, pleural, soft tissue, spleen, subcutis, thyroid gland and renal.

**Table 8:** Cox Regression Analysis for Overall Survival for Patients Who Received LRT

Characteristics		Univariate Analysis		Multivariate Analysis	
		Hazard Ratio (95% CI)	*p*-Value	Hazard Ratio (95% CI)	*p*-Value
Age (ref: <75 y)	≥75 y	1.5 (0.9–2.3)	0.17	0.9 (0.5–1.7)	0.89
Sex (ref: male)	Female	0.6 (0.4–0.8)	**0.002**	0.7 (0.4–1.1)	0.09
WHO-PS (ref: 0–1)	2–3	2.1 (1.1–4.0)	**0.03**	1.5 (0.7–3.2)	0.30
Smoking (ref: never)	Former	1.1 (0.1–7.1)	0.65		
	Current	1.9 (0.7–4.7)	0.18		
PD-L1 status (ref: >50% expression)	1%–49% expression	2.1 (1.3–3.4)	0.06	1.9 (1.0–3.7)	0.05
	<1% expression	2.0 (1.2–3.4)	**0.01**	1.5 (0.8–2.8)	0.24
	Unknown	2.1 (1.3–3.4)	**0.002**	1.6 (0.9–2.7)	0.10
Histology (ref: nonsquamous)	Squamous	1.1 (0.7–1.6)	0.77		
Brain imaging (ref: MRI)	Diagnostic CT-scan	1.3 (0.9–1.8)	0.24		
T Stage (ref: 1-2)	3–4	1.1 (0.8–1.6)	0.63		
N Stage (ref: 0-1)	2–3	1.1 (0.7–1.6)	0.74		
Total metastases (ref: 1)	>1	0.9 (0.6–1.3)	0.55		
Metastatic sites					
(ref: no adrenal gland)	Adrenal gland	1.7 (0.9–2.9)	0.05		
(ref: no bone)	Bone	1.6 (1.0–2.6)	0.08		
(ref: no brain)	Brain	1.5 (0.5–1.0)	0.05		
(ref: no extrathoracic nodal)	Extrathoracic nodal	0.8 (0.5–1.3)	0.38		
(ref: no lung)	Lung	1.1 (0.7–1.6)	0.76		
(ref: no other^a^)	Other site	1.2 (0.7–2.0)	0.54		
Serum albumin (ref: <40 g/L)	≥40 g/L	1.4 (0.8–2.3)	0.22		
Serum CRP (ref: ≤5 mg/L)	>5 mg/L	2.1 (1.3–3.6)	**0.01**	2.2 (1.3–3.7)	**0.003**
Serum LDH (ref: <248)	≥248	0.8 (0.6–1.2)	0.36		
Best response to induction systemic therapy (ref: CR and PR)	SD	2.0 (1.4–2.8)	**<0.001**	1.7 (1.1–2.7)	**0.02**
Immunotherapy (ref: No)	Yes	0.4 (0.2–0.7)	**<0.001**	0.3 (0.1–0.6)	**0.002**
TRAE (ref: toxicity ≤ 2)	Toxicity grade ≥ 3	2.4 (0.8–7.7)	0.13		

*Note:p* values in bold are statistically significant.

CI, confidence interval; CR, complete response; CRP, C-reactive protein; CT, computed tomography; LDH, lactate dehydrogenase; LRT, local radical therapy; MRI, magnetic resonance imaging; PD-L1, programmed death-ligand 1; PD, progressive disease; PR, partial response; Ref, reference; SD, stable disease; TRAE, treatment related adverse event; WHO-PS, WHO Performance Score.

a Bowel, breast, liver, pancreatic gland, peritoneal, pleural, soft tissue, spleen, subcutis, thyroid gland and renal.

## Discussion

We described in a large, adequately staged, and homogeneous cohort that the use of ICI as an induction strategy results in improved survival in patients with sOMD NSCLC, regardless of LRT. This was found despite a higher incidence of N3 disease and a higher number of metastases in the (chemo-)ICI group. Furthermore, the addition of LRT did not substantially increase the percentage of TRAE in patients treated with an ICI-based strategy, confirming our previously published preliminary monocenter data.^20^ Given that, regardless of the systemic treatment strategy, a relevant percentage (47%–65%) of patients did not proceed to LRT, our study stresses the selection bias in LRT studies only enrolling patients without disease progression on systemic therapy.^3^^,^^4^^,^^6^^,^^15^^,^^17^^,^^19^ Outcomes of these trials probably overestimate the survival of patients with synchronous oligometastatic NSCLC, as those not proceeding to LRT (because of disease progression, toxicity of induction treatment, or LRT not possible owing to disease burden) have a worse prognosis and are not taken into account in these studies. Indeed, our study data suggest that patients not proceeding to LRT do worse. Given that no response to induction therapy was independently linked to poor survival, this underscores the urgency for the selection of better systemic therapy strategies. Our results also suggest that patients with a response to an ICI-based strategy can obtain a durable response (with or without LRT, as patients with a CR did not proceed to LRT), given that there is a plateau in the tail of the OS curve in patients treated with (chemo-)ICI compared with the chemotherapy-only group.

Currently, patients are selected for LRT for OMD on the basis of the number and location of metastases. As most patients will not benefit from adding LRT, our results stress the need for biomarkers for patient selection and biomarker-based personalized treatment strategies.^18^ Established (PD-L1 expression) and promising (i.e., tumor mutational burden, circulating tumor DNA, imaging-based) biomarkers for ICI treatment in advanced NSCLC are available, but data specifically for sOMD NSCLC are limited.^18^^,^^21^, ^22^, ^23^ The factors associated with survival from our study are in line with the results of multiple first-line phase 3 (chemo-)ICI studies in advanced NSCLC, for example, an ICI-based regimen was associated with prolonged survival outcomes, and no response (stable disease or progressive disease) to induction systemic therapy and elevated levels of serum CRP were associated with shorter survival.^24^, ^25^, ^26^, ^27^, ^28^, ^29^, ^30^ The factors associated with survival in the group of patients with intention of LRT and those who actually proceeded to LRT are similar, except for the presence of brain metastases, which was not associated with poor OS in those receiving LRT. Other possible prognostic factors, such as PD-L1 expression, total number of metastases, smoking status, and N stage, were not associated with survival outcomes, emphasizing the need for larger future prospective trials to identify true prognostic factors for patients with sOMD NSCLC.^24^, ^25^, ^26^, ^27^, ^28^, ^29^, ^30^ Interestingly, the presence of bone metastases was not associated with reduced survival in the subgroup treated with LRT, whereas in the metastatic setting, bone metastases are associated with poor outcomes.^31^^,^^32^ LRT could counteract the immunosuppressive tumor microenvironment typically found in bone metastases.^33^

Our results are promising for an ICI-based treatment strategy in sOMD and are similar to the results for ICI treatment in early and advanced NSCLC.^24^, ^25^, ^26^, ^27^, ^28^, ^29^, ^30^^,^^34^ However, several unanswered questions remain regarding the optimal strategy to combine LRT and ICI in sOMD NSCLC. It is unknown whether patients with a disease response on induction ICI should continue ICI after LRT, and if so, the duration of adjuvant treatment. This will be evaluated in the phase 2 and 3 ICARS study (NCT06219317). The best sequence of treatments (LRT followed by ICI, or ICI followed by LRT) is also unknown. As potential micrometastases are undetected by contemporary staging techniques, it seems logical to start with systemic therapy to eradicate these micrometastases and to avoid the potential toxicity of LRT in those nonresponsive to systemic therapy.^14^ However, upfront LRT seems feasible as the median PFS and OS in a single arm phase 2 study evaluating pembrolizumab after LRT in oligometastatic NSCLC were similar to our data.^19^ Of note, survival outcomes were only evaluated in those starting pembrolizumab (45 of 51), and 31 of 45 patients had metachronous metastases, which are associated with better outcomes compared with synchronous metastases.^15^^,^^19^ Although upfront systemic therapy reduces the potential of micrometastases, systemic therapy comes with potential toxicity and this toxicity could be avoided or delayed by upfront LRT.^35^

Our results imply that an ICI-based treatment is beneficial in sOMD NSCLC, but they do not answer the question of whether these patients actually need LRT, or that ICI by itself outperforms the benefit of LRT, as has been suggested by the NRG-LU002 trial (NCT03137771).^36^ This randomized phase 2 and 3 trial was prematurely closed after interim analysis of the phase 2 part. It was evaluated whether adding LRT to maintenance systemic therapy improved PFS (phase 2) and OS (phase 3) in patients with oligometastatic NSCLC (≤ 3 extracranial metastases, n = 185) with at least stable disease after induction systemic therapy. Approximately 90% of the patients had received ICI. There were no significant differences in PFS for the total (HR = 0.93, 95% CI: 0.65–1.31) and the ICI subgroup (HR = 0.90, 95% CI: 0.61–1.32), suggesting that LRT does not influence PFS and even causes additional toxicity. Limitations were the following: (1) PET-CT and brain imaging were not mandatory for initial staging; and (2) metastases were counted after induction therapy, making it unclear whether patients with true sOMD were enrolled although the median number of metastases was two (range 1-25, quartiles 1-3, 2-3). Patients with metachronous metastases were eligible, further limiting the interpretation of the data. However, cohort one of the multicenter phase 2 ETOP-CHESS trial (NCT03965468, N = 47, n = 42 efficacy analysis), including only adequately staged patients with synchronous oligometastatic NSCLC (≤3 metastases, of which one metastasis should be extracranial), was also negative for its primary end point, PFS rate at 12 months. In this trial, the efficacy of an induction strategy with concurrent durvalumab, chemotherapy, and SRT to all oligometastatic sites followed by definitive radiotherapy or surgery to the primary for patients responding to induction therapy was investigated.^37^ Although the strategy was safe, only 13 of 42 patients in the efficacy analyses were without disease progression after 1 year, which was below the predefined threshold (50%). More than 25% of patients had progression of disease early in the induction phase, again implying the need for further improvement of the systemic therapy regimen. Cohort two (addition of tremelimumab to durvalumab) is currently enrolling and both cohorts have extensive translational research parts to identify patients benefiting from these treatment strategies, and results are awaited. Another ongoing phase 3 randomized trial (SARON, NCT02417662) evaluates whether adding SRT (all disease sites) to standard systemic therapy (including ICI) improves survival compared with standard systemic therapy in adequately staged patients with sOMD NSCLC (≤5 metastases in ≤3 organs).^38^

Strong points of our study are that we present the largest series to date, including only adequately staged NSCLC regardless of treatment (intention-to-treat). By screening all MDTs, we detected all consecutive patients, as all new NSCLC diagnoses have to be discussed in the MDT. The retrospective nature with its inherent biases was a limitation. In addition, the chemotherapy-only group was dominant given that, for most of these patients, first-line (chemo-)ICI was not available in the Netherlands at the time of diagnosis (2017: first-line monotherapy ICI available, 2019 first-line chemo-ICI available), whereas others were ineligible because of ICI contraindications (such as autoimmune disease or high-dosage of corticosteroids). Selection bias regarding these treatments could not be prevented. Furthermore, the PD-L1 expression level was unbalanced between the groups (higher percentage of patients with high PD-L1 expression in the (chemo)-ICI group) which could have influenced our outcomes, as high PD-L1 expression is associated with better outcomes on ICI).^21^^,^^39^, ^40^, ^41^ In addition, high PD-L1 expression seems to be associated with poor survival in patients not treated with ICI, as high expression by tumors leads to evading the immune system, which, in turn, could influence survival in the chemotherapy-only group.^42^, ^43^, ^44^ Of note, patients in the chemotherapy-only group had significantly more patients with unknown PD-L1 expression, which is probably owing to the earlier part of the inclusion period in which testing for PD-L1 expression was not routine practice.

## Conclusions

For patients with sOMD NSCLC, an ICI-based systemic treatment strategy (±LRT) is safe and associated with improved survival compared with a chemotherapy-only (±LRT) strategy. As a significant percentage of patients do not proceed to LRT because of disease progression, improved systematic therapies are needed. Prospective randomized trial data, including potential biomarkers, are needed to help identify patients who are most likely to benefit from adding LRT.

## CRediT Authorship Contribution Statement

**Mandy Jongbloed:** Acquisition of data, Statistical analysis, Writing – original draft preparation, Writing – review & editing.

**Valentina Bartolomeo:** Acquisition of data, Writing – review & editing.

**Martina Bortolot:** Writing – review & editing.

**Shahan Darwesh:** Writing – review & editing.

**Jarno W. J. Huijs:** Writing – review & editing.

**Safiye Dursun:** Acquisition of data.

**Juliette Degens:** Writing – review & editing.

**Ben E. E. M. van den Borne:** Writing – review & editing.

**Maggy Youssef-El Soud:** Writing – review & editing.

**Marcel Westenend:** Writing – review & editing.

**Cordula Pitz:** Writing – review & editing.

**Dirk K. M. De Ruysscher:** Conceptualization Statistical analysis, Writing – review & editing, Supervision.

**Lizza E. L. Hendriks:** Conceptualization, Acquisition of data, Statistical analysis, Writing – original draft preparation, Writing – review & editing, Supervision.

All authors have read and agreed to the published version of the manuscript.

## Disclosure

Dr. Hendriks: outside of this manuscript personal fees as an invited speaker from AstraZeneca, Bayer, Eli Lilly, Merck Sharp & Dohme, high5oncology, Takeda, Janssen, GlaxoSmithKline, Sanofi, Pfizer (Inst), Medtalks, Benecke, VJOncology, Medimix (self).; all payments were paid to the institution with the exception of Medtalks, Benecke, VJOncology, Medimix; fees paid to her institution for advisory board membership from Advisory boards: Amgen, Boehringer Ingelheim, Eli Lilly, Novartis, Pfizer, Takeda, Merck, Janssen, Merck Sharp & Dohme, Anheart, Bayer, AZ, Pierre Fabre, Bristol-Myers Squibb, AbbVie, Daiichi; institutional research grants from 10.13039/100004337Roche 10.13039/100004328Genentech, 10.13039/100004325AstraZeneca, Boehringer Ingelheim, Takeda, Merck, Pfizer, Novartis, and Gilead; institutional funding as a local principal investigator from AstraZeneca, GlaxoSmithKline, Novartis, Merck, Roche, Takeda, Blueprint, Mirati, AbbVie, Gilead, Merck Sharp & Dohme, Merck, Amgen, Boehringer Ingelheim, Pfizer; Member guideline committees: Dutch guidelines on NSCLC, brain metastases and leptomeningeal metastases (self), ESMO guidelines on metastatic NSCLC and SCLC (nonfinancial) Other (nonfinancial): secretary NVALT studies foundation, subchair European Organization for Research and Treatment of Cancer metastatic NSCLC systemic therapy, vice-chair scientific committee Dutch Thoracic Group. Dr. de Ruysscher: outside of this manuscript research grant/support/Advisory Board: Institutional financial interests (no personal financial interests) from AstraZeneca, Bristol-Myers Squibb, BeiGene, Philips, Olink and Advisory Board: Institutional financial interests (no personal financial interests) for Eli Lilly. The remaining authors declare no conflict of interest.
