# Supplementary material for: Impact of Immune Checkpoint Inhibitors and Local Radical Treatment on Survival Outcomes in Synchronous Oligometastatic NSCLC
Source: JTO Clin Res Rep. 2025 Jan 8;6(3):100790. doi: 10.1016/j.jtocrr.2025.100790 (PMC11847110; doi:10.1016/j.jtocrr.2025.100790)
Supplement: Supplementary Tables 1-3 [file mmc1.docx]

**Supplemental tables**

**Supplementary table 1. Description and frequency of local radical treatment (LRT) of all patients treated with induction systemic therapy.**

|  | *Procedure* |  |
| --- | --- | --- |
| **Surgery** | **Adrenal gland resection** | 9 (23%) |
|  | **Breast ablation** | 1 (2%) |
|  | **Corpectomy of thoracic vertebrae** | 1 (2%) |
|  | **Liver metastasis resection** | 2 (5%) |
|  | **Lobectomy + lymph node dissection** | 15 (39%) |
|  | **Omentectomy (1 lesion)** | 1 (2%) |
|  | **Pneumonectomy** | 1 (2%) |
|  | **Resection brain metastasis** | 9 (23%) |
|  | **Small bowel resection** | 1 (2%) |
| **Radiation therapy** | ***VMAT primary tumor and thoracic lymph nodes*** | 145 (89%) |
|  | Radiation therapy dose | 61.1 ± 4.4 Gy (55-69Gy) |
|  | Number of fractions | 24.5 ± 3.4 (20-33) |
|  | ***SBRT primary tumor*** | 18 (11%) |
|  | Radiation therapy dose | 48.0 ± 2.6 Gy (40-55Gy) |
|  | Number of fractions | 4.7 ± 2.1 (4-11) |
|  | ***VMAT metastases*** | 60 (36%) |
|  | Radiation therapy dose | 60.9 ± 3.8 Gy (54-69Gy) |
|  | Number of fractions | 23.5 ± 3.1 (20-33) |
|  | ***SBRT metastases*** | 107 (64%) |
|  | Radiation therapy dose | 31.1 ± 11.9 Gy (16-60Gy) |
|  | Number of fractions | 3.3 ± 3.1 (1-17) |

Abbreviations: VMAT; Volumetric Modulated Arc Therapy, SBRT; stereotactic body radiation therapy.

**Supplementary table 2. Toxicities in patients with sOMD NSCLC with the intention of radical treatment treated with (chemo-)ICI or chemotherapy only**

| **Toxicity** | **Grade 1** | | **Grade 2** | | **Grade 3** | | **Grade 4** | | **Grade 5** | |
| --- | --- | --- | --- | --- | --- | --- | --- | --- | --- | --- |
|  | ***(Chemo-)ICI*** | ***Chemo only*** | ***(Chemo-)ICI*** | ***Chemo only*** | ***(Chemo-)ICI*** | ***Chemo only*** | ***(Chemo-)ICI*** | ***Chemo only*** | ***(Chemo-)ICI*** | ***Chemo only*** |
| **Acute kidney injury** |  |  |  |  | 2 | 5 |  |  |  |  |
| **Allergic reaction** |  |  |  | 1 | 5 |  |  |  |  |  |
| **Alopecia** |  |  |  | 1 |  |  |  |  |  |  |
| **Anemia** |  |  |  | 1 |  | 23 | 1 | 2 |  |  |
| **Arterial thromboembolism** |  |  |  | 1 |  |  |  |  |  |  |
| **Arthritis** |  |  | 1 |  |  |  |  |  |  |  |
| **Atrial fibrillation** |  |  |  | 1 |  |  |  |  |  |  |
| **Atrial flutter** |  |  |  | 1 |  |  |  |  |  |  |
| **Bone pain** |  |  |  |  |  | 2 |  |  |  |  |
| **Bronchial obstruction** |  |  |  |  |  |  | 1 |  |  |  |
| **Bronchopleural fistula** |  |  | 1 |  |  |  |  |  |  |  |
| **Bronchopulmonary hemorrhage** |  |  |  | 1 |  |  | 1 |  |  |  |
| **Cerebral edema** |  |  |  |  | 1 | 1 |  |  |  |  |
| **Chest pain** |  |  |  | 1 |  | 1 |  |  |  |  |
| **Cognitive disturbance** |  |  |  |  |  | 1 |  |  |  |  |
| **Colitis** |  |  | 2 |  |  |  | 1 |  |  |  |
| **Constipation** |  |  |  |  |  | 1 |  |  |  |  |
| **Cystitis** |  | 1 | 1 |  |  |  |  |  |  |  |
| **Diarrhea** |  | 2 | 1 |  | 2 | 4 |  |  |  |  |
| **Dry eye** | 1 |  |  |  |  |  |  |  |  |  |
| **Duodenal ulcer** |  |  |  |  |  | 1 |  |  |  |  |
| **Dyspnea** |  |  |  | 1 | 1 |  |  |  |  |  |
| **Eczema** |  |  | 1 |  |  |  |  |  |  |  |
| **Enterocolitis** |  |  |  |  |  |  |  |  |  | 1 |
| **Epistaxis** | 1 | 1 |  |  |  |  |  |  |  |  |
| **Fatigue** | 1 | 2 |  | 2 |  | 1 |  |  |  |  |
| **Febrile neutropenia** |  |  | 1 | 2 | 3 | 12 |  | 1 |  | 2 |
| **Fever** |  | 1 | 1 | 1 | 2 | 5 |  | 1 |  |  |
| **Gastritis** |  |  |  |  |  | 1 |  |  |  |  |
| **Gingival pain** |  |  |  | 1 |  |  |  |  |  |  |
| **Guillan-Barre syndrome** |  |  |  |  |  |  | 1 |  |  |  |
| **Hearing impaired** |  |  |  | 1 |  | 2 |  |  |  |  |
| **Heart failure** |  |  |  |  |  | 1 | 1 | 1 |  | 1 |
| **Hepatitis** |  |  |  | 1 | 5 |  |  |  |  |  |
| **Herpes simplex reactivation** |  |  |  | 1 |  |  |  |  |  |  |
| **Hyperthyroidism** | 1 |  |  |  |  |  |  |  |  |  |
| **Hypokalemia** |  |  |  |  | 1 |  |  |  |  |  |
| **Hyponatremia** |  |  |  |  |  |  |  | 1 |  |  |
| **Hypotension** |  |  |  |  |  | 1 |  |  |  |  |
| **Hypothyroidism** |  |  | 5 |  |  |  |  |  |  |  |
| **Ischemia cerebrovascular** |  |  |  |  |  | 1 |  |  |  |  |
| **Laryngitis** |  |  |  | 1 |  |  |  |  |  |  |
| **Liver infection** |  |  | 1 |  |  |  |  |  |  |  |
| **Lung infection** |  |  | 2 | 2 | 4 | 7 |  |  |  | 1 |
| **Malaise** |  |  | 2 | 3 | 4 | 8 |  | 1 |  |  |
| **Mucositis oral** |  |  | 1 | 1 |  |  |  |  |  |  |
| **Myocardial infarction** |  |  |  |  |  |  | 1 | 1 |  |  |
| **Nausea** | 1 | 1 |  | 9 |  | 5 |  |  |  |  |
| **Palmar-plantar erythrodysesthesia syndrome** |  |  | 1 |  |  |  |  |  |  |  |
| **Peripheral motor neuropathy** |  |  | 1 |  |  |  |  |  |  |  |
| **Peripheral sensory neuropathy** | 1 |  | 3 |  | 2 |  |  |  |  |  |
| **Pleural effusion** |  |  |  | 1 |  |  |  |  |  |  |
| **Pleural infection** |  |  |  |  |  | 1 |  |  |  |  |
| **Pneumonitis** |  |  | 3 | 1 | 3 |  |  |  | 1 |  |
| **Pneumothorax** |  |  | 1 | 1 |  | 1 |  |  |  |  |
| **Radiation dermatitis** |  |  | 1 |  |  |  |  |  |  |  |
| **Radiation esophagitis** |  |  | 1 | 2 | 2 | 4 |  | 1 |  |  |
| **Radiation pneumonitis** |  |  | 2 | 5 | 1 | 3 |  | 1 |  |  |
| **Rash maculo-papular** |  |  | 1 |  | 1 |  |  |  |  |  |
| **Rash pustular** | 2 |  | 3 | 2 | 1 |  |  |  |  |  |
| **Shingles** |  |  | 1 |  |  |  |  |  |  |  |
| **Subcutaneous emphysema** |  |  |  |  |  | 1 |  |  |  |  |
| **Thromboembolic event** |  |  | 1 | 3 | 3 | 6 |  |  |  |  |

**Supplementary table 3. Toxicities in patients treated with (chemo-)ICI**

| **Grading** | **(chemo-)ICI without LRT (n=101)** | **(chemo-)ICI and LRT (n=37)** | **P value** |
| --- | --- | --- | --- |
| **1** | 6 (6%) | 2 (6%) | 0.91 |
| **2** | 24 (24%) | 15 (41%) | 0.05 |
| **3** | 32 (32%) | 11 (30%) | 0.83 |
| **4** | 4 (4%) | 3 (8%) | 0.33 |
| **5** | 1 (1%) | 0 (0%) | 0.54 |
